# Lack of Association between Missense Variants in *GRHL3* (rs2486668 and rs545809) and Susceptibility to Non-Syndromic Orofacial Clefts in a Han Chinese Population

**DOI:** 10.1371/journal.pone.0159940

**Published:** 2016-07-26

**Authors:** Miao He, Zhuan Bian

**Affiliations:** 1 State Key Laboratory Breeding Base of Basic Science of Stomatology (Hubei-MOST) & Key Laboratory of Oral Biomedicine Ministry of Education, School & Hospital of Stomatology, Wuhan University, Wuhan, China; 2 Department of Pediatric Dentistry, School & Hospital of Stomatology, Wuhan University, Wuhan, China; University of Kentucky, UNITED STATES

## Abstract

**Background:**

Grainyhead-like-3 (*GRHL3*) was recently identified as the second gene that, when mutated, can leads to Van der Woude syndrome, which is characterized by orofacial clefts (OFC) and lower lip pits. In addition, a missense variant (rs41268753) in *GRHL3* confers risk for non-syndromic cleft palate cases of European ancestry. Together with interferon regulatory factor 6 (*IRF6*), *GRHL3* may be associated with the risk of NSOFC which awaits for being verified across different ethnic populations.

**Objective:**

The aim of this study was to investigate the possible relationship between common functional variants in *GRHL3* and susceptibility to NSOFC, especially cleft palate cases, in a Han Chinese population, one of the ethnic groups with the highest birth prevalence of orofacial clefting.

**Methods:**

Because the allele frequency for rs41268753 minor alleles was zero in our Chinese population, we selected functional single nucleotide polymorphisms (SNPs) spanning *GRHL3* with minor allele frequencies (MAFs) > 5% in the Han Chinese population. Two SNPs which meet the above criteria were then genotyped in a case-control cohort comprising 1145 individuals using the TaqMan 5′-exonuclease allelic discrimination assay.

**Results:**

SNPs rs2486668 and rs545809 were used in this study. Overall genotype and allele distributions of both SNPs in general and stratified genotyping analyses revealed no statistically significant differences between cases and controls. Further logistic regression analyses using different genetic models failed to reveal any evidence that these markers influence risk to NSOFC.

**Conclusions:**

The variant rs41268753 in *GRHL3* increases the risk for cleft palate in European population, but our findings failed to detect the link between two *GRHL3* SNPs (rs2486668 and rs545809) and risk to NSOFC in the Han Chinese cohort. Although the present study did not provide any evidence that common functional variants in *GRHL3* may contribute to NSOFC etiology in this Chinese population, further studies with a larger sample size, additional SNPs, and a more diverse ethnic cohort are still warranted.

## Introduction

Orofacial clefts (OFC) are among the most common congenital defects with a relatively high birth prevalence of 1.66/1000 newborns in China [1]. Categorization according to anatomical site shows that OFCs are commonly subtyped as cleft lip only (CLO), cleft lip with palate (CLP), or cleft palate only (CPO). Given their shared epidemiological traits and embryologic origin, CLO and CLP are traditionally combined as a single subgroup, namely, cleft lip with or without cleft palate (CL/P) [2]. Nevertheless, an increasing number of evidence indicates CLO and CLP might have different genetic origins and would be better analyzed as distinct entities [3]. Most OFCs occur as non-syndromic (NS) cases where clefting occurs as the only malformation in the affected infant, and are thought to be caused by the interplay between environmental and genetic factors [4]. Although previous studies of linkage and candidate genes, and more recently, several genome-wide association studies (GWAS), have reported multiple risk loci associated with NSOFC [5–10], the underlying genetic architecture of this birth defect remains largely unknown.

A powerful molecular approach with which to study the genetics of NSOFC is to investigate causative genes for syndromic clefting. Interferon regulatory factor 6 (*IRF6*) is one such candidate gene, and mutations in *IRF6* cause Van der Woude syndrome (VWS, [MIM 119300]), the most common type of syndromic OFC, or popliteal pterygium syndrome (PPS, [MIM 119500]) [11]. Furthermore, variants in *IRF6* show a strong association with NSOFC, especially with the CLO subtype [12]. However, findings from different studies have been inconsistent and the risk association of NSOFC with particular variants of *IRF6* may differ depending on ethnic background [13–15]. Thus far, mutations in *IRF6* have been identified in only 70% of families with VWS. A linkage study for a large Finnish pedigree with VWS identified a novel locus on 1p33–p36 (VWS2) rather than *IRF6* at 1q32–q41, providing further evidence of locus heterogeneity underlying this syndrome [16]. Further mutation screening in VWS families without *IRF6* mutation demonstrated that *GRHL3* is the second reported gene for which mutations resulting in VWS. Phenotypic analyses of murine embryos with double heterozygous knockout (*Irf6*^+/-^; *Grhl3*^+/-^) indicated that both genes play pivotal roles in the development of the functional periderm, and disturbing this process leads to VWS [17]. Functional analyses also showed *Grhl3* is a key downstream target of *Irf6* in the process of periderm differentiation [18]. Taken together, previous studies demonstrate mutations in both *IRF6* and *GRHL3* cause almost the same clefting phenotypes. Furthermore, the recent studies by GWAS and sequencing approaches have indicated a missense variant (rs41268753) in *GRHL3* increases risk for NSCPO cases of European ancestry [10, 19]. However, the allele frequency for rs41268753 minor alleles is zero in Chinese population according to the same report and the Hapmap database. More recently, an association study genotyped multiple tag single nucleotide polymorphisms (SNPs) spanning the *GRHL3* gene in Chinese NSCL/P patients and controls and had not reached a clear-out conclusion that any genotype or haplotype variant is associated with the susceptibility to NSCL/P in this cohort [20]. Hence, we examined other common functional variants in *GRHL3* (rather than rs41268753 itself) involved in the pathogenesis of NSOFC in a Chinese Han population. The aim of this study was to investigate any possible association between *GRHL3* polymorphisms and susceptibility to NSOFC in this Han Chinese population.

## Materials and Methods

### Study population and DNA samples

The Ethics Committee of School of Stomatology, Wuhan University, Wuhan, China (protocol number: 2014–20) has approved this study. All applicable regulations concerning the use of human DNA were followed. A cohort comprising 768 cases, and 377 controls, was used for genotyping and all subjects were ethnic Han Chinese. Any associated anomalies or syndromes were excluded by two experienced surgeons and only those diagnosed with NSOFC were thus included. All participants or their guardians have signed the informed consent forms. Peripheral venous blood samples were collected and genomic DNA was extracted as previously described [9].

### Selection of *GRHL3* polymorphisms

Functional SNPs for *GRHL3* located in either the coding region or the regulatory elements (5’-flanking region and 5’/3’-untranslated regions) with the minor allele frequency (MAF) > 5% in Han Chinese population were selected from the International HapMap Project database (http://hapmap.ncbi.nlm.nih.gov), and the National Center for Biotechnology Information (NCBI) database (http://www.ncbi.nlm.nih.gov/projects/SNP).

### Genotyping

The TaqMan 5′-exonuclease allelic discrimination assay (Applied Biosystems, Foster City, CA) was used to perform the genotyping. Technicians were blinded to the case or control status of individuals when performing it. Finally, genotyping was randomly replicated for 10% of samples to ensure genotypic consistency.

### Statistical analyses

The Hardy-Weinberg equilibrium (HWE) was evaluated for both polymorphisms in the control group. Statistical analyses of case *vs*. control status was performed using the SPSS package to test the null hypothesis of independence between disease and marker genotypes (SPSS Inc., Chicago, IL, USA). Comparison of genotype and allele frequencies among cases, and the control group, were performed by the Chi-square test. Odds ratios (ORs) with 95% confidence intervals (CI) were computed from unconditional logistic regression analyses. Pairwise linkage disequilibrium (LD) was indicated as both Lewontin D′ and r^2^ values by use of the Haploview program (http://www.broad.mit.edu/mpg/haploview/index.php/).

## Results

### Characteristics of the genotyped cohort

The case and control groups were satisfactorily matched for gender (P > 0.05). According to clinical manifestations, 768 NSOFC patients were divided into four main subgroups: 297 CPO patients and 471 CL/P patients, which were further subdivided into 214 CLO patients and 257 CLP patients, respectively.

### SNP data

Both selected tag SNPs (rs2486668 and rs545809) in *GRHL3* are described in Table 1. These were successfully genotyped, with a call rate > 99% (S1 and S2 Figs, S1 Table). Again, 10% of samples were randomly selected for duplicate genotyping, which resulted in completely concordant results. The genotypic frequencies of each SNP conformed to the Hardy–Weinberg equilibrium (both P > 0.05) among controls, indicating there is no population stratification present in this study.

**Table 1:** General information for genotyped SNPs.

SNP ID	Gene	Chr.	Posotion	Region	SNPs	Function	Amino Acid Changes	MAF^a^ (CHB)	HWE^b^ (P-value)
rs2486668	*GRHL3*	1	24658063	exon 2	C>G	missense	Asp^c^→Glu^d^	0.22	0.996
rs545809	*GRHL3*	1	24690764	exon 16	T>A	missense	Met^e^→ Lys^f^	0.42	0.997

^a^MAF, minor allele frequency; CHB, Chinese Han Chinese in Beijing, China.

^b^Hardy-Weinberg equilibrium among controls.

^c^Asp, aspartic acid.

^d^Glu, glutamic acid.

^e^ Met, methionine.

^f^ Lys, lysine.

### Distribution of genotypic and allele frequencies

The MAFs of the tested SNPs in the controls were similar to those recorded in the HapMap CHB cohort. As listed in Table 2, the frequencies of the rs2486668 CC, CG, and GG genotypes (62.5%, 33.2% and 4.3%, respectively) in both controls and NSOFC cases were identical (P = 0.994). Significant differences were not observed in allele frequencies between groups. Further logistic regression analyses unraveled that neither the CG heterozygote, nor the GG homozygote, conferred to NSOFC risk when comparing with the wild-type homozygote (OR = 1.00, 95% CI = 0.77–1.30 for CG and OR = 1.01, 95% CI = 0.54–1.87 for GG, respectively). Analyses using different genetic models also uncovered no differences in genotype distribution between NSOFC cases and controls. Given that genetic etiologies may be diverse in different subtypes of NSOFC, a stratified analysis was also conducted for CL/P, CLP, CLO, and CPO, respectively. We observed that for rs2486668, overall genotype and allele frequencies for the subgroups differed slightly from those in controls. However, the differences were not statistically significant between controls and each subgroup, and a lack of any significant association was also observed in logistic regression analyses under these different models of inheritance.

**Table 2:** Genotypic and allelic distributions of rs2486668 in NSOFC cases and controls.

rs2486668	Controls (n = 376,%)	All cleft cases (n = 768,%)	CL/P (n = 471, %)	CLP (n = 257, %)	CLO (n = 214, %)	CPO (n = 297, %)
**Genotype**						
CC	235(62.5)	480(62.5)	290(61.6)	161(62.6)	129(60.3)	190(64.0)
CG	125(33.2)	255(33.2)	157(33.3)	85(33.1)	72(33.6)	98(33.0)
GG	16(4.3)	33(4.3)	24(5.1)	11(4.3)	13(6.1)	9(3.0)
P^a^	-	0.994	0.8428	0.999	0.5963	0.694
**OR(95% CI)**						
CG vs. CC	-	1.00(0.77, 1.30)	1.02(0.80, 1.30)	0.99(0.72, 1.38)	1.05(0.71, 1.55)	0.97(0.66, 1.41)
GG vs. CC	-	1.01(0.54, 1.87)	1.22(0.70,1.80)	1.00(0.48, 2.10)	1.48(0.64, 3.41)	0.70(0.29, 1.68)
CG/GG vs. CC	-	1.00(0.78, 1.29)	1.04(0.82,1.32)	0.99(0.73, 1.36)	1.09(0.76, 1.60)	0.93(0.65, 1.35)
CG/CC vs. GG	-	0.99(0.54, 1.82)	0.83(0.48, 1.41)	0.99(0.48, 2.06)	0.69(0.30, 1.57)	1.42(0.60, 3.39)
**Alleles**						
C	0.79	0.79	0.78	0.79	0.77	0.8
G	0.21	0.21	0.22	0.21	0.23	0.2
P^a^	-	1	0.8592	0.9538	0.8989	0.8431

CL/P, cleft lip with or without cleft palate; CLP, cleft lip with palate; CLO, cleft lip only; CPO, cleft palate only; OR, odds ratio; CI, confidence interval.

^a^Comparison of genotype and allele frequencies among cases and controls were performed by two-sided Chi-square test.

Similar data were also observed for rs545809 (Table 3). We could not identify any relationship between rs545809 and the risk of NSOFC using general or stratified analyses. In the LD analyses, we found no strong linkage disequilibrium (D’ = 0.177; r^2^ = 0.571) between the two SNPs (rs2486668 and rs545809). Given the lack of association with single marker analyses, haplotypes of these two markers would not differ between cases and controls. Therefore, haplotype analyses were not undertaken here.

**Table 3:** Genotypic and allelic distributions of rs545809 in NSOFC cases and controls.

rs545809	Controls (n = 376,%)	All cleft cases (n = 768,%)	CL/P (n = 471, %)	CLP (n = 257, %)	CLO (n = 214, %)	CPO (n = 297, %)
**Genotype**						
TT	139(37.0)	284(37.0)	157(33.3)	94(36.6)	63(29.4)	127(42.8)
AT	180(47.9)	368(47.9)	235(50.0)	117(45.5)	118(55.1)	133(44.8)
AA	57(15.2)	116(15.1)	79(16.8)	46(17.9)	33(15.4)	37(12.5)
P^a^	-	0.9997	0.7195	0.7782	0.4741	0.1575
**OR(95%CI)**						
AT vs. TT	-	1.00(0.76, 1.31)	1.16(0.90, 1.49)	0.96(0.69, 1.35)	1.45(0.96, 2.18)	0.81(0.55, 1.20)
AA vs. TT	-	1.00(0.68, 1.45)	1.23(0.87, 1.73)	1.19(0.77, 1.86)	1.28(0.74, 2.21)	0.71(0.41, 1.24)
AT/AA vs. TT	-	1.00(0.77, 1.29)	1.17(0.92, 1.49)	1.02(0.74, 1.40)	1.41(0.95, 2.07)	0.79(0.54, 1.14)
AT/TT vs. AA	-	1.00(0.71, 1.42)	0.89(0.65, 1.21)	0.82(0.55, 1.22)	0.98(0.60, 1.60)	1.26(0.76, 2.08)
**Alleles**						
T	0.61	0.61	0.58	0.59	0.57	0.65
A	0.39	0.39	0.42	0.41	0.43	0.35
P^a^	-	1	0.3772	0.5578	0.4725	0.0769

CL/P, cleft lip with or without cleft palate; CLP, cleft lip with palate; CLO, cleft lip only; CPO, cleft palate only; OR, odds ratio; CI, confidence interval.

^a^Comparison of genotype and allele frequencies among cases and controls were performed by two-sided Chi-square test.

## Discussion

NSOFC is a complex malformation, thought to be of multifactorial etiology, with both genetic and environmental elements. This complexity has impeded the process of screening causal genetic risk factors [4]. In the past few decades, candidate gene-based approaches have proven to be a useful tool with which to investigate the genetic components of NSOFC. However, thus far, only *IRF6* has shown any convincing degree of consistency across studies [3, 12,13]. *GRHL3* was recently identified as a second causative gene for VWS, providing a prominent candidate susceptible gene for NSOFC [17]. Furthermore, a missense variant in *GRHL3* was identified to be associated with the risk for NSCPO in European population [10, 19]. A recent association study was performed in Chinese NSCL/P patients using ten tag SNPs of *GRHL3* and did not give an affirmative answer that this gene contributes to NSCL/P etiology in the Chinese Han population [20]. Therefore, it is necessary to further investigate whether there are other common functional variants in *GRHL3* involved in the pathogenesis of NSOFC, especially NSCPO, in the Chinese population.

Grainyhead genes encode a well conserved family of transcription factors including three members, Grhl1, 2, and 3 in mice, which have ubiquitous expression patterns in the process of embryonic development, and in adult skin cells. Despite sharing extensive sequence homology, overlapping expression patterns, and a consensus in DNA binding sequence, *Grhl3* is indispensable in the courses of neural tube closure, skin barrier formation and wound healing [21]. *Grhl3* is directly regulated by *Irf6*, but functions in independent, but convergent pathways during palatogenesis [17, 18]. In *Irf6* deficient mice, orofacial clefting was caused by abnormal periderm differentiation and a failed disappearance of the medial edge epithelium (MEE). Conversely, the MEE was successfully cleared in *Grhl3*^*-/-*^ embryos. Thus the common feature of *Irf6* and *Grhl3* mutants may be a failed periderm development, providing further confirmation of a role for the oral periderm in orofacial development. Mutations in the *IRF6* and *GRHL3* genes can lead to VWS, with a dysfunctional oral periderm contributing to the phenotypes of orofacial clefts in humans [17].

Although locus heterogeneity for VWS was further confirmed, we also noted that the deleterious mutations in *GRHL3* may not be prevalent in VWS pedigrees without mutations in *IRF6* [17]. Sequencing of the coding regions of the *GRHL3* gene in three Chinese VWS-affected families lacking *IRF6* mutations identified no new pathogenic mutations (unpublished data). In the mouse, *Grhl3*^*-/-*^ embryos with an abnormal oral periderm developed cleft palate at low penetrance (17%) [17]. Potential causative genes for VWS awaits being identified in the future. So far, the precise biological role of *Grhl3* in facial morphogenesis is still elusive. In vivo functional dissection of *GRHL3* missense variants will be performed in zebrafish models in our future study. In addition, identification of the downstream targets of the Irf6-Grhl3 network will help not only to enrich our knowledge of craniofacial development, but also to provide more candidates involved in the genetic etiology of syndromic and/or non-syndromic clefting in humans.

The results presented in the current study are frustrating, although not entirely unexpected given the heterogeneous nature of NSOFC. First, population differences may affect the results of genetic association study in complex diseases. For example, inconsistent results in *IRF6* association studies have been reported using patient cohorts derived from distinct regions within China [13–15]. The common variant (rs41268753) in the *GRHL3* locus identified by the recent GWAS and sequencing is associated with the risk to NSCPO in European population, but not in several Asian and African-derived populations [10, 19]. Further well-designed studies including diverse ethnic backgrounds are now warranted. Second, we adopted stratified analyses in this study in which CL/P and CPO were analyzed separately as distinct entities.The VWS-affected patients with *GRHL3* mutations were reported to have a higher proportion of having CP than those with *IRF6* mutations [17]. The results from GWAS and sequencing have also found the SNP (rs41268753) in *GRHL3* is associated with NSCPO, but not with NSCL/P [10, 19]. The association analysis in the Chinese CL/P patients identified two SNPs and a haplotype of *GRHL3* that reached the significance level, but none of them survived the multiple comparisons [20]. Third, the limited sample size may also have affected the results of our study. However, the number of NSCPO cases in the current study is comparable to the recent GWAS that found the *GRHL3* hit [10]. Meanwhile, the MAFs of both SNPs for controls from this study and from publicly-available databases are very similar, indicating favorable quality control. Therefore, increasing the sample size is unlikely to have any effect. Lastly, considering more markers as haplotypes be a more efficient analytic tool in this situation than the study of one or two SNPs at the population level [22]. However, the original purpose of this study was to investigate the potential link between common functional variants in *GRHL3* and risk to NSOFC in the Han Chinese population. Only two SNPs met the selection criteria and were used in this study. Given the lack of association with single marker analyses, it is now imperative to conduct additional SNP-based haplotype analyses and re-sequencing studies in the future.

In summary, this preliminary study investigated whether *GRHL3* was involved in the pathogenesis of non-syndromic clefting in the Han Chinese population. Our findings failed to detect two missense variants (rs2486668 and rs545809) in *GRHL3* contribute to NSOFC risk in Han Chinese cases and controls. More cohort-based studies with wider SNP coverage of the *GRHL3* locus, recruiting from different ethnic populations, are still warranted to further verify any relationship between *GRHL3* and risk to NSOFC.

## Supporting Information

S1 Fig Representative fluorescence scatter plot for rs2486668 in Taqman assay. (JPG)

S2 Fig Representative fluorescence scatter plot for rs545809 in Taqman assay. (JPG)

S1 Table Genotype counts for rs2486668 and rs545809 in the sample set. (XLS)

## References

[1] Dai L, Zhu J, Mao M, Li Y, Deng Y, Wang Y, et al Time trends in oral clefts in Chinese newborns: data from the Chinese National Birth Defects Monitoring Network. Birth Defects Res A Clin Mol Teratol 2010; 88: 41–47. 10.1002/bdra.20607 19691087 PMC3110751

[2] Dixon MJ, Marazita ML, Beaty TH, Murray JC. Cleft lip and palate: understanding genetic and environmental influences. Nat Rev Genet 2011;12: 167–178. 10.1038/nrg2933 21331089 PMC3086810

[3] Rahimov F, Marazita ML, Visel A, Cooper ME, Hitchler MJ, Rubini M, et al Disruption of an AP-2alpha binding site in an IRF6 enhancer is associated with cleft lip. Nat Genet 2008; 40: 1341–1347. 10.1038/ng.242 18836445 PMC2691688

[4] Shi M, Christensen K, Weinberg CR, Romitti P, Bathum L, Lozada A, et al Orofacial cleft risk is increased with maternal smoking and specific detoxification-gene variants. Am J Hum Genet 2007;80: 76–90. 17160896 10.1086/510518 PMC1785306

[5] Marazita ML, Murray JC, Lidral AC, Arcos-Burgos M, Cooper ME, Goldstein T, et al Meta-analysis of 13 genome scans reveals multiple cleft lip/palate genes with novel loci on 9q21 and 2q32-35. Am J Hum Genet 2004; 75: 161–173. 15185170 10.1086/422475 PMC1216052

[6] Birnbaum S, Ludwig KU, Reutter H, Herms S, Steffens M, Rubini M, et al Key susceptibility locus for nonsyndromic cleft lip with or without cleft palate on chromosome 8q24. Nat Genet 2009; 41: 473–477. 10.1038/ng.333 19270707

[7] Mangold E, Ludwig KU, Birnbaum S, Baluardo C, Ferrian M, Herms S, et al Genome‐wide association study identifies two susceptibility loci for nonsyndromic cleft lip with or without cleft palate. Nat Genet 2010; 42: 24–26. 10.1038/ng.506 20023658

[8] Beaty TH, Murray JC, Marazita ML, Munger RG, Ruczinski I, Hetmanski JB, et al A genome‐wide association study of cleft lip with and without cleft palate identifies risk variants near MAFB and ABCA4. Nat Genet 2010; 42: 525–529. 10.1038/ng.580 20436469 PMC2941216

[9] Sun Y, Huang Y, Yin A, Pan Y, Wang Y, Wang C, et al Genome-wide association study identifies a new susceptibility locus for cleft lip with orwithout a cleft palate. Nat Commun 2015; 6: 6414 10.1038/ncomms7414 25775280

[10] Leslie EJ, Liu H, Carlson JC, Shaffer JR, Feingold E, Wehby G, et al A genome-wide association study of nonsyndromic cleft palate identifies an Etiologic Missense Variant in GRHL3. Am J Hum Genet 2016; 98: 744–754. 10.1016/j.ajhg.2016.02.014 27018472 PMC4833215

[11] Kondo S, Schutte BC, Richardson RJ, Bjork BC, Knight AS, Watanabe Y, et al Mutations in IRF6 cause Van der Woude and popliteal pterygium syndroms.Nat Genet 2002; 32: 285–289. 12219090 10.1038/ng985 PMC3169431

[12] Zucchero TM, Cooper ME, Maher BS, Daack-Hirsch S, Nepomuceno B, Ribeiro L, et al Interferon regulatory factor 6 (IRF6) gene variants and the risk of isolated cleft lip or palate. N Engl J Med 2004; 351: 769–780. 15317890 10.1056/NEJMoa032909

[13] Huang Y, Wu J, Ma J, Beaty TH, Sull JW, Zhu L, et al Association between IRF6 SNPs and oral clefts in West China. J Dent Res 2009; 88: 715–718. 10.1177/0022034509341040 19734457 PMC2901597

[14] Shi J, Song T, Jiao X, Qin C, Zhou J. Single-nucleotide polymorphisms (SNPs) of the IRF6 and TFAP2A in non-syndromic cleft lip with or without cleft palate (NSCLP) in a northern Chinese population. Biochem Biophys Res Commun 2011; 410: 732–736. 10.1016/j.bbrc.2011.06.006 21683068

[15] Paranaíba LM, Bufalino A, Martelli-Júnior H, de Barros LM, Graner E, Coletta RD. Lack of association between IRF6 polymorphisms (rs2235371 and rs642961) and non-syndromic cleft lip and/or palate in a Brazilian population.Oral Dis 2010; 16: 193–197. 10.1111/j.1601-0825.2009.01627.x 19780991

[16] Koillinen H, Wong FK, Rautio J, Ollikainen V, Karsten A, Larson O, et al Mapping of the second locus for the Van der Woude syndrome to chromosome 1p34. Eur J Hum Genet 2001; 9: 747–752. 11781685 10.1038/sj.ejhg.5200713

[17] Peyrard-Janvid M, Leslie EJ, Kousa YA, Smith TL, Dunnwald M, Magnusson M, et al Dominant mutations in GRHL3 cause Van der Woude Syndrome and disrupt oral periderm development. Am J Hum Genet 2014; 94: 23–32. 10.1016/j.ajhg.2013.11.009 24360809 PMC3882735

[18] de la Garza G, Schleiffarth JR, Dunnwald M, Mankad A, Weirather JL, Bonde G, et al Interferon regulatory factor 6 promotes differentiation of the periderm by activating expression of Grainyhead-like 3. J Invest Dermatol 2013; 133: 68–77. 10.1038/jid.2012.269 22931925 PMC3541433

[19] Mangold E, Bohmer AC, Ishorst N, Hoebel AK, Gultepe P, Schuenke H, et al Sequencing the GRHL3 coding region reveals rare truncating mutations and a common susceptibility variant for nonsyndromic cleft palate. Am J Hum Genet 2016; 98: 755–762. 10.1016/j.ajhg.2016.02.013 27018475 PMC4833214

[20] Wang Y, Sun Y, Huang Y, Pan Y, Jia Z, Ma L, et al Association study between Van der Woude Syndrome causative gene GRHL3 and nonsyndromic cleft lip with or without cleft palate in a Chinese cohort. Gene 2016; 588: 69–73. 10.1016/j.gene.2016.04.045 27129939

[21] Boglev Y, Wilanowski T, Caddy J, Parekh V, Auden A, Darido C, et al The unique and cooperative roles of the Grainy head-like transcription factors in epidermal development reflect unexpected target gene specificity. Dev Biol 2011; 349: 512–522. 10.1016/j.ydbio.2010.11.011 21081122

[22] Kerameddin S, Namipashaki A, Ebrahimi S, Ansari-Pour N. IRF6 is a marker of Severity in Nonsyndromic Cleft Lip/Palate. J Dent Res 2015; 94: 226S–232S. 10.1177/0022034515581013 25896061

