# Supplementary figures and images for: Lack of Association between Missense Variants in GRHL3 (rs2486668 and rs545809) and Susceptibility to Non-Syndromic Orofacial Clefts in a Han Chinese Population
Source: PLoS One. 2016 Jul 26;11(7):e0159940. doi: 10.1371/journal.pone.0159940 (PMC4961390; doi:10.1371/journal.pone.0159940)

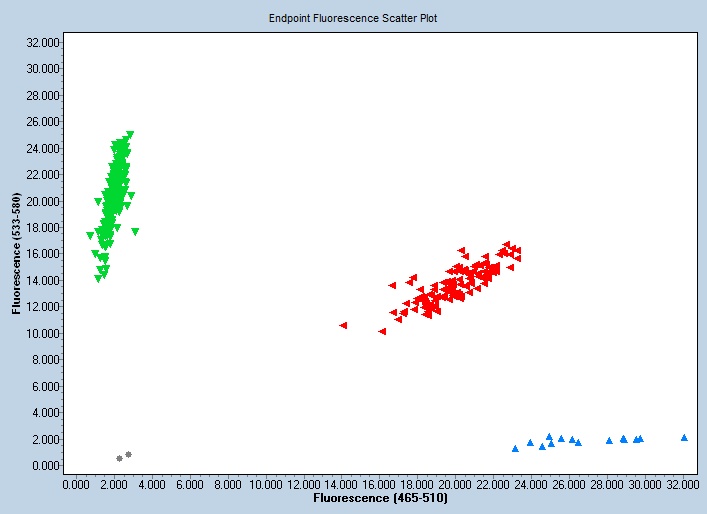

Supplement: S1 Fig — (JPG) [file pone.0159940.s001.jpg]

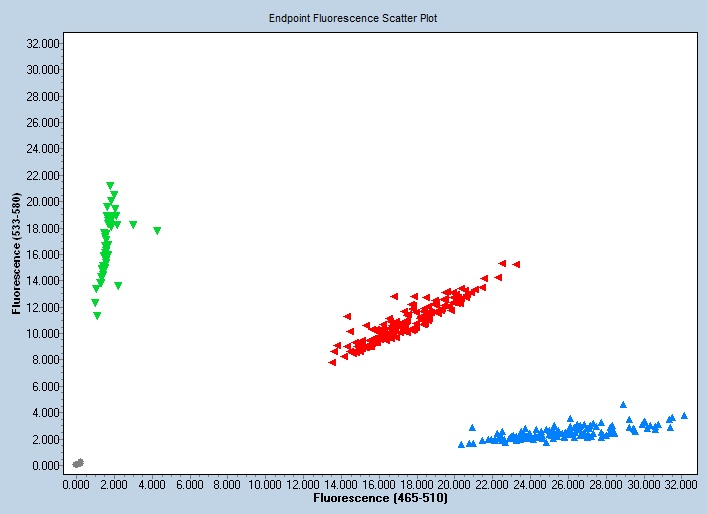

Supplement: S2 Fig — (JPG) [file pone.0159940.s002.jpg]
